# Surface Plasmon Resonance: A Versatile Technique for Biosensor Applications

**DOI:** 10.3390/s150510481

**Published:** 2015-05-05

**Authors:** Hoang Hiep Nguyen, Jeho Park, Sebyung Kang, Moonil Kim

**Affiliations:** 1 BioNanotechnology Research Center, Korea Research Institute of Bioscience and Biotechnology (KRIBB), Daejeon 305-806, Korea; E-Mails: hhiep86@kribb.re.kr (H.H.N.); jayho88@kribb.re.kr (J.P.); 2 Department of Nanobiotechnology, Korea University of Science and Technology (UST), Daejeon 305-350, Korea; 3 Department of Biological Sciences, School of Life Sciences, Ulsan National Institute of Science and Technology (UNIST), Ulsan 689-798, Korea; 4 Department of Pathobiology, College of Veterinary Medicine Nursing & Allied Health (CVMNAH), Tuskegee University, Tuskegee, AL 36088, USA

**Keywords:** surface plasmon resonance, SPR, biosensor, SPR imaging, applications

## Abstract

Surface plasmon resonance (SPR) is a label-free detection method which has emerged during the last two decades as a suitable and reliable platform in clinical analysis for biomolecular interactions. The technique makes it possible to measure interactions in real-time with high sensitivity and without the need of labels. This review article discusses a wide range of applications in optical-based sensors using either surface plasmon resonance (SPR) or surface plasmon resonance imaging (SPRI). Here we summarize the principles, provide examples, and illustrate the utility of SPR and SPRI through example applications from the biomedical, proteomics, genomics and bioengineering fields. In addition, SPR signal amplification strategies and surface functionalization are covered in the review.

## 1. Introduction

Numerous strategies for protein labeling have been developed that allow the characterization of proteins regarding their structure, folding or interaction with other proteins [1,2]. Labeling strategies are used to bring about the covalent attachment of reporter tags, such as biotin, radioisotopes, fluorophores, or enzymes, to the target biomolecules (*i.e.*, proteins and nucleotides) to quantitatively assess binding among biomolecules [3,4]. In addition, the use of molecular labels can cause steric hindrance or change structural configurations, affecting the labelled molecules’ affinities for their target biomolecules, which is a major challenge. Label-free detection eliminates the need for specialized tags or dyes, thereby allowing the sensitive measurement of target analytes and enabling the use of native biomolecules suitable for biologically relevant approaches. In the past few decades, a variety of optical biosensor methods have been developed, including surface plasmon resonance (SPR) [5], quartz crystal microbalance (QCM) [6], and ellipsometry [7]. Among the various optical sensing methodologies, the SPR-based system is a representative type of label-free technique for monitoring biomolecular interactions in real-time.

Since it was first introduced in the early 1990s, SPR has been proven to be one of the most powerful technologies to determine specificity, affinity and kinetic parameters during the binding of macromolecules in many bonds types, including protein-protein [8,9], protein-DNA [10,11], enzyme-substrate or inhibitor [4,12], receptor-drug [13,14], lipid membrane-protein [15,16], protein-polysaccharide [17], cell or virus-protein [18,19,20], among others. This optical technique measures the refractive index changes in the vicinity of thin metal layers (*i.e.*, gold, silver, or aluminum films) in response to biomolecular interactions. Before a sample solution flows across the SPR surface, capturing agents, such as antibodies, enzymes, peptides and DNAs are immobilized on the surface. The changes in the SPR angle, which is the angle of minimum reflectivity, can be determined by varying the incidence angle and recording the reflected light intensity during the biological binding reactions between various biomolecules. So far, numerous studies have advanced the potential of SPR sensors by increasing the effectiveness of the technique [21,22,23]. Accordingly, the possible fields of application of SPR technology have expanded to biomedical, environmental and industrial areas. As has been extensively and intensively documented in the literature, SPR is an acceptable method for disease diagnosis, drug discovery, foodborne pathogen detection, and so on [24,25]. Above all, the application of SPR for biomedical purposes is remarkable. So far, various types of SPR measurement systems have been developed for the monitoring of chemical and biological species via the basic theory of SPR detection [26,27,28]. Here we review recent advances in SPR biosensing, with a particular focus on practical applications of SPR-type biosensors, describing their usefulness and challenges for bioassays.

## 2. Operating Principle of SPR Biosensors

### 2.1. General Principle of SPR

**Figure 1:**
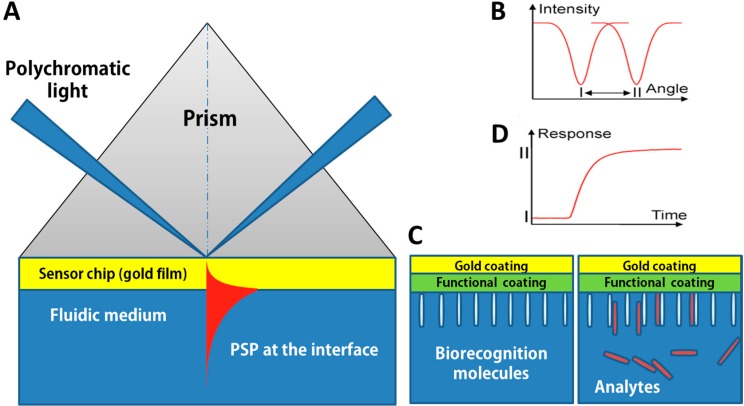
Concept of a surface plasmon resonance (SPR) biosensor: (**A**) Kretschmann geometry of the ATR method; (**B**) spectrum of reflected light before and after refractive index change; (**C**) analyte-biorecognition elements binding on SPR sensor surface and (**D**) refractive index changes caused by the molecular interactions in the reaction medium. Adapted from [30].

Surface plasmon resonance occurs when a photon of incident light hits a metal surface (typically a gold surface). At a certain angle of incidence, a portion of the light energy couples through the metal coating with the electrons in the metal surface layer, which then move due to excitation. The electron movements are now called plasmon, and they propagate parallel to the metal surface. The plasmon oscillation in turn generates an electric field whose range is around 300 nm from the boundary between the metal surface and sample solution [29]. In a commercial SPR biosensor configuration, incident light is employed by using a high-reflective index glass prism in the Kretschmann geometry of the attenuated total reflection (ATR) method (Figure 1). The defined SPR angle, at which resonance occurs, on the conditions of the constant light source wavelength and metal thin surface, is dependent on the refractive index of the material near the metal surface. Consequently, when there is a small change in the reflective index of the sensing medium (e.g., through biomolecule attachment), plasmon cannot be formed. Detection is thus accomplished by measuring the changes in the reflected light obtained on a detector. In addition, the amount of surface concentration can be quantified by monitoring the reflected light intensity or tracking the resonance angle shifts. Typically, an SPR biosensor has a detection limit on the order of 10 pg/mL.

In SPR biosensors, probe molecules are firstly immobilized on to the sensor surface. When the solution of target molecules is flown into contact with the surface, a probe-target binding via affinity interaction occurs, which consequently induces an increase in the refractive index at the SPR sensor surface (Figure 1D). In SPR experiments, resonance or response units (RU) are used to describe the signal change, where 1 RU is equivalent to a critical angle shift of 10^−4^ degree. At the start of the experiment whereas probe target interactions have not occurred, the initial RU value corresponds to the starting critical angle. The change in refractive index Δn_d_ arisen within a layer of thickness h can be calculated as

Δn_d_ = (dn/dc)_vol_ ΔΓ/h (1)

where (dn/dc)_vol_ is the increase of refractive index n with the volume concentration of analyte c, and ΔΓ is the concentration of the bound target on the surface [31]. The change in the refractive index is tracked by the coupling of incident light into a propagating surface plasmon (PSP) on a gold surface in real time. Accordingly, the rate of association (k_on_) during association phase, the rate of disassociation (k_off_) when target molecules is removed from the continuous flow by buffer washing, and the association rate constant (k_a_) and dissociation rate constant (k_d_) can be determined by SPR evaluation of binding kinetics. The parameter related to the refractive index can also be used to detect and quantify the target molecules bound to a known probe immobilized on the sensor surface. The limit of detection (LOD) in the SPR experiment depends on a number of factors including the molecular weight, optical property and binding affinity of target-probe molecules as well as the surface coverage of the probe molecule.

### 2.2. SPR Imaging Principle

**Figure 2:**
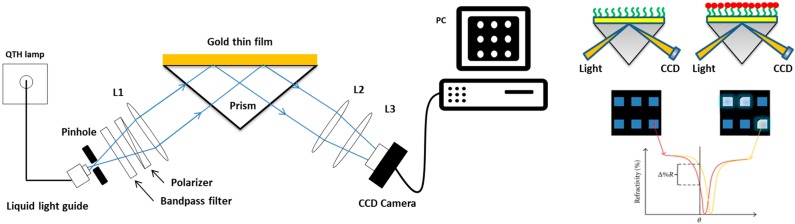
General principle of surface plasmon resonance imaging (SPRI). (**Left**) The instrumentation of an SPR imaging system: The light source is a quartz tungsten-halogen lamp; the light is delivered through a liquid light guide to a goniometer arm, collimated by lenses, and passed through a narrow interference filter and a polarizer. A *p*-polarized and monochromatic light beam is then focused directly onto a prism coupler. The reflected light from the gold surface is captured by a monochromatic CCD camera. L2, L3 are lenses positioned in front of CCD for higher quality images. The images could be digitally stored using a B/W frame grabber and further analyzed using photography software; (**Right**) The analyte-ligand interaction shifts the SPR curve towards a higher angle (red to orange). Due to the measurement confinements (fixed wavelength and angle of incidence θ), changes in the reflectivity (Δ%R) at a single spot of the array can be simultaneously detected. Adapted from [32].

In high-throughput screenings and multiplex analyses, a combination of protein arrays and the SPR technique would provide an excellent alternative and label-free method in comparison with the existing methods that require kinetic data. However, standard SPR biosensor instrumentation, with 3–4 flow cells on a single sensor chip, acts more as a hindrance than an aid to high-throughput screening (HTS) applications. To overcome this obstacle, a modified version of SPR design, called SPR imaging (SPRI), has been developed to simultaneously process hundreds or thousands of samples. SPR imaging systems using rapid optical arrays have potential applications in the high-throughput screening of drugs and biomarkers, as well as clinical diagnosis through their use in multi-array detection systems. SPR imaging technology represents an important step forward to SPR analysis. Not eliminating the sensitive label-free method, SPRI’s additional value lies in its capability to visualize a whole biochip via a CCD camera. The improved design enables biochips to be prepared in an array format, in which each array spot can provide a wealth of SPR information simultaneously. As shown in Figure 2, in an SPRI system, a coherent polarized light beam is used instead of polychromatic light. This change helps expand the light cover on a larger area of the sensing surface. The reflected light is captured by a charge-coupled device (CCD) camera for further imaging analysis. The high resolution CCD camera provides images across the array format in real-time with up to hundreds of active spots. Captured images that show local changes at the chip surface can provide detailed information on molecular binding, interactions or kinetic processes. Unlike conventional SPR, the measurement conducted by SPRI is stringently performed at a constant wavelength and a constant angle [32]. Thus, changes in reflected light intensity are proportional to any variation of the refractive index near the metal surface [33].

## 3. Applications of SPR-Based Biosensors

### 3.1. Biomedical Applications

SPR biosensing appears to be one of the most powerful approach for monitoring of affinity binding of biomolecules, and primary screening of druggable molecules. SPR-type sensors are increasingly used to study a variety of biological entities, such as DNAs, RNAs, proteins, carbohydrates, lipids, and cells in the field of biomedical research. In this subsection, several examples of biomedical applications of SPR technology including interaction analyses, conformational change studies, and mutation detection are described.

#### 3.1.1. Interaction Analyses

A wide range of applications has been developed for the use of SPR biosensors in the biomedical field. First of all, SPR has been used as a powerful tool to study interactions between biomolecules based on affinity binding analysis of a variety of bonds, including antibody-antigen [34], ligand-receptor kinetics [35,36,37,38,39,40,41,42], enzyme-substrate reaction [4,43] and epitope mapping [44]. Real-time monitoring of DNA manipulation such as hybridization kinetics, enzymatic modifications, and DNA strand separation using SPR biosensors was earlier introduced in 1995 by Nilsson *et al.* [45]. The advent of click chemistry has allowed scientists to design DNA analogs with novel properties unseen in nature as well as improved stability, functionality and binding characteristics that can be utilized to develop innovative therapeutic agents or new tools for diagnostics. Artificial nucleosides with unusual structural features, such as peptide nucleic acid (PNA), locked nucleic acid (LNA), hexitol nucleic acid (HNA) and phosphoramidates morpholino (MORF) oligomers have proven advantages over functional nucleic acids (aptamers and DNAzymes) in terms of denaturation and biodegradation stability in body fluids. In SPR studies, aptamers are considered promising recognition elements with good chemical stability, high selectivity and high affinity toward their targets, and they are easily chemically modified. Aptamers offer more advantages than antibodies. SPR detection was employed in the selection of an RNA aptamer for human influenza [46], and aptamer-based SPR analyses were successfully applied in the detection of human IgE [47], C-reactive protein (CRP) [48] and the HIV-1-trans-activating (Tat) protein [49], and RBP4 (retinol binding protein 4), a diabetes biomarker [50]. There is another branch of DNA analogs that are designed to target single-stranded DNA and RNA with high affinity and specificity; they are conformationally restricted DNA analogs, such as PNA, LNA, HNA and MORF. These molecules have great uses in radiopharmaceutical applications. Many researchers have utilized these artificial molecules to study DNA hybridization [51,52], pathogen DNA detection, single-nucleotide polymorphisms (SNPs) [53] and miRNA detection. A thorough review of the use of these DNA analogs as recognition elements in SPR-based sensing can be found elsewhere [54].

#### 3.1.2. Conformational Change Studies

In addition, the SPR signal intensity has been shown to be strongly affected by optical thickness changes in the sensor metal film, as well as by refraction index changes taking place near the metal surface (~200 nm). As a protein molecule undergoes a structural change, those optical indicators are also affected and can be monitored by SPR biosensors. Nevertheless, the SPR technique is often used as a complementary method to verify conformational changes study rather than as a primary technique. This application of the SPR technique has been used to monitor structural transition in protein-small molecule interactions [12], proteins under diverse environmental conditions [55,56] or impacts on apoptosis inducers [57]. In an attempt to detect protein conformational changes, in 2005, Kim *et al.* developed an antibody chip with conformational specificity to the Bax protein, a pro-apoptotic member of the Bcl-2 family of proteins, which plays a pivotal role in the mitochondrial pathway for apoptosis [57]. Bax conformational change was first induced by the administration of an apoptosis inducer, TNF-related apoptosis-inducing ligand (TRAIL) and then measured by SPRI. The results indicated that only structurally altered Bax gave visible SPR images, while intact Bax seldom showed any data.

#### 3.1.3. Mutation Detection

Another extension of SPR-based detection applications is its use in point mutation detection by combining SPR with other conventional techniques. For example, an SPR biosensor was utilized for the detection of point mutation using polymerization extension reaction [58]. In this experiment, the capture DNA and probe complementary DNA were coated by a natural complement, and PCR reaction was carried out directly on-chip. Only wild type DNA showed signal boosting by PCR, while mutant DNA showed no SPR signal amplification. Mutation in protein molecules has also been studied using the SPR technique [59]. The DNA-binding capability of tumor protein p53 was evaluated. This protein is the “master switch” for the control of cell proliferation, whose mutation causes genetic alterations in human cancers. DNA was immobilized on a BIACORE CM5 chip for the protein binding experiment. Purified wild p53 and mutant p53 (R248W) were injected at a concentration of 100 nM and a flow rate of 5 μL∙min^−1^ for 6 min. The SPR data showed that the RU value of the mutant p53 protein was 4.7 times lower than that of wild p53 protein in response to 100 nM of each protein. Based on the fact that the SPR response is directly proportional to the amount of p53 protein that interacted with the consensus DNA coated on the gold thin film, the results obtained from SPR sensorgrams demonstrated that the wild p53 protein could bind to the cognate DNA sequence while mutant p53 protein could not.

### 3.2. High-Throughput Screening (HTS)

**Figure 3:**
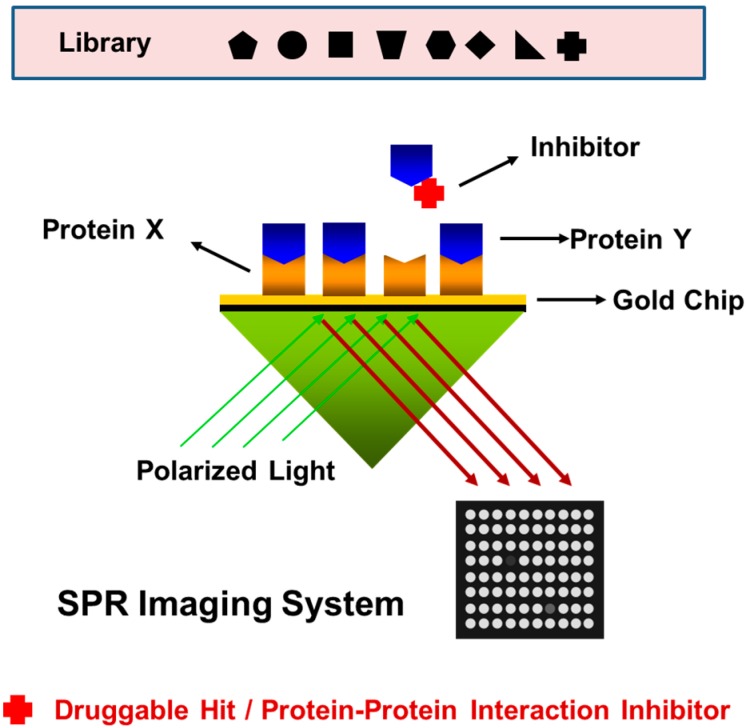
High-throughput drug screening using an SPR imaging protein chip system. The bright image indicates protein-protein interaction on a gold surface. Upon the binding of an inhibitor to the target protein, protein-protein interactions are disrupted, resulting in changes in SPR imaging signal intensity and a darker image. Adapted from [62].

The application extensions of SPR biosensors are not only limited to ligand-receptor interaction kinetics dynamic analyses; they are also used for drug discovery and drug development. There are several different formats of SPR biosensors, including the array format, multi-channel unit format, and SPR imaging format, which allow simultaneous and continuous detection to analyze the performance of hundreds to thousands of affinity binding events on a chip surface [60,61]. In SPR imaging, the incidence angle remains fixed, and the binding of biomolecules on a gold surface is measured as the change in reflectivity (or reflectance) in relation to the incident ray intensity, unlike SPR sensors that depend on the measurement of the absorption dip in the SPR angle or SPR wavelength. Despite the excellent benefits inherent in SPR technology, conventional propagating SPR biosensors have a serious limitation in their inability to support multiplex analysis, as less than four analyses with conventional SPR instruments make such parallel operations feasible. In contrast, SPR imaging technology using a multi-analyte biosensor permits a high-throughput approach, and it achieves a similar degree of sensitivity that achieved by conventional SPR biosensors (Figure 3) [62]. Thus, SPR imaging systems without any labeling requirements are more suitable for high-throughput screening (HTS), particularly in drug discovery, than any other optics-based detection techniques [63].

Krishnamoorthy *et al.* used a commercial SPR imaging instrument for the screening of kinetics and affinity constants of multiple biomolecular interactions simultaneously on a 6 × 4 microarray chip [64]. Ligands were serially diluted and spotted on the array following by an analyte sample injection. The SPR signal responses of 24 microarray spots were collected and used as data for the rates and affinity constant calculations. This method significantly reduces experiment time and cost in comparison with traditional approaches. In 2004, Maillart *et al.* described an SPR imaging system-based HTS approach for multiple analyses of interactions between the p53 transcription factor and its corresponding cis-acting DNA elements [65]. In 2007, Neuman *et al.* developed a method capable of detecting up to 9216 target-ligand interactions on a single array [66].

### 3.3. Proteomics Researches

In the field of proteomics, methods for the identification and analysis of biomarkers have gained more attention during recent years and have evolved rapidly. The identification and detection of disease biomarkers are important to foresee outbreaks of certain diseases, thereby avoiding surgery and other invasive and expensive medical treatments for patients. Thus, more research into discovering new biomarkers and new methods for faster and more accurate detection is needed. It is often difficult to detect and measure biomarkers because of their low concentrations and the complexity of their respective matrices. Therefore, it is hard to find and validate accurate screening methods suitable for clinical use. SPR has proven to be useful in biomarkers diagnosis due to its sensitivity, portability, obviation of large sample volumes, and capability of multiplexed detection. SPR biosensors have been successfully applied in detecting the biomarkers of numerous diseases, such as breast, ovarian, and pancreatic cancer, as well as cardiac and neurological diseases.

#### 3.3.1. Tumor-Associated DNA Markers

In 2006, Li *et al.* developed a combination of surface hybridization, surface ligation and nanoparticle amplification for single-nucleotide polymorphism (SNP) genotyping in the BRCA1 gene [67], which is one of the two major genes (BRCA1 and BRCA2) reported to be connected to breast cancer susceptibility. These two tumor suppressor genes are involved in repairing the DNA double-strand breaks that are responsible for breast cancer. Mutations in these two genes can lead to instability of the human genome and can increase the risk of breast cancer for future patients from the age of 30 [68]. Therefore, the identification of BRCA mutations is of a great relevance for prospective interventions and treatments for breast cancer patients. By using nanoparticles with oligonucleotides complementary to the ligation probe DNA aiming at enhancing the SPR signal, single mismatches of BRCA1 were successfully detected at concentrations as low as 1 pM. The nanoparticles substantially help overcome the limitation of conventional SPR biosensors as, without amplification, the SPR direct detection limit is about 100 nM [69]. Label-free detection is simpler than label techniques; thus, a minimum detectable concentration for mutation analysis without labeling is relevant to optimize SPR methodology. Another commonly mutated gene in human cancers is the tumor suppressor p53 gene, whose main function is to mediate either cell cycle arrest or apoptosis. The loss of its activation is required for new oncogenic activities to promote tumorigenesis and drug resistance. It is reported that more than 50% of cancer patients show p53 gene mutation [70]. In another study, both wild-type and mutant p53 proteins at SPR chip preimmobilized with consensus DNA and monoclonal antibody were detected [71]. The normal cell samples displayed significantly higher levels of wild-type p53; therefore, they showed higher affinity to the immobilized consensus double-stranded DNA. Meanwhile, the pre-immobilized monoclonal antibodies showed a specific affinity for the total p53. The difference between the SPR signals revealed the extent of p53 mutation. Low detection levels of wild-type p53 (10.6 pM) and total p53 (1.06 pM) were obtained due to the strong affinity of the consensus ds-DNA to the wild-type p53 and that of the antibody to total p53.

#### 3.3.2. Disease-Related Protein Biomarkers

**Figure 4:**
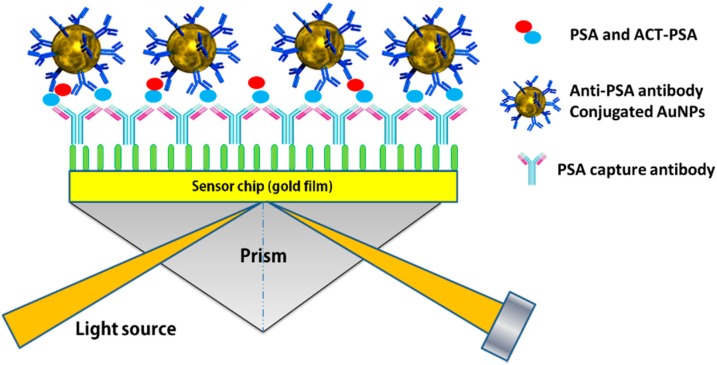
Schematic representation of the PSA immunosensor based on PSA detection antibody-modified Au nanoparticles using surface plasmon resonance (SPR). For the PSA sandwich analysis, following PSA/tPSA bindingto the SPR sensor layer, 1.5 μg∙mL^−1^ PSA detection antibody or Anti-PSA antibody conjugated AuNPs (20 nm, 5.1 × 1010 nanoparticles/mL or 40 nm, 5.1 × 1010 nanoparticles/mL) was injected on the surface. Adapted from [72].

In contrast to genetic markers, protein markers enable rigorous evaluation for clinical applications; therefore, protein-based assays are being developed. Because biomarkers in real blood samples exist in small concentration, this hinders the use of SPR biosensors. To overcome this, the aid of nanoparticles is needed for signal amplification. With the assistance of nanoparticles, researchers have been able to employ SPR in the detection of important biomarkers, such as total prostate-specific antigen (tPSA) [72], carbohydrate antigen 15-3 (CA15-3) [73], carcinoembryonic antigen (CEA) [74], C-reactive protein (CRP) [75], human epidermal growth factor receptor 2 (HER2) [76], estrogen receptor (ER) [14,77,78], progesterone receptor (PR) [79,80], *etc*. Figure 4 shows the schematic representation of PSA sandwich assay using PSA detection antibody-modified AuNPs [72]. By using 40 nm nanoparticles conjugated with the PSA antibody, a tPSA assay was performed on 75% human serum at a detection limit of 0.29 ng∙mL^−1^ (8.5 pM); tPSA was obtained in the 75% serum, which corresponds to 0.39 ng∙mL^−1^ in whole serum with the SPR sensor. As this detection limit is lower than the threshold value for prostate cancer detection (2.5 ng∙mL^−1^), it is possible to clinically diagnose prostate cancer with the developed immunoassay. Short assay time, repeated usability of the same sensor chip, ability to detect PSA in high serum concentration, and real-time testing enable the application of the optimized assay as a point-of-care device for clinical prostate cancer diagnosis and prognosis.

CEA is the human cancer-associated antigen and serologic tumor biomarkers for diagnosis of colorectal, gastric and pancreatic cancers. The CEA level is a commonly used index in treatment after breast cancer surgery to identify recurrences. The normal CEA level is in the range of 3−5 ng/mL for a healthy woman. In 2006, Tang *et al.* used an SPR-based immunosensor that used protein A conjugated on a gold surface [74]. Next, a monoclonal anti-CEA antibody was coupled to protein A and prepared for CEA injections. The detection limit of 0.5 ng/mL was obtained for the purified CEA. C-reactive protein (CRP) is a principal blood serum biomarker for conventional inflammation and low-grade inflammation diagnosis. CRP is produced by the liver and rises in level whenever inflammation occurs throughout the body. Jung and coworkers used the CRP monoclonal antibody and an amide-linked (AL) N-hydroxysuccinimide (NHS)−dextran functionalized gold array surface in a spectral SPR system to detect C-reactive protein (CRP) in human sera with up to 120 human sera [75].

### 3.4. Cellular Analysis and Cell-Based Detection

The SPR technique is not only advantageous due to its real-time and label-free imaging capabilities for dynamic changes at the surface but also for cellular changes, such as physiology, interactions at the cell-surface, and cell detection. The SPR technique enables the monitoring of cellular response, cellular adhesion and cellular products as well as the detection of cancer cells and bacteria cells .Upon receiving stimulation from reactive molecules, mammalian cells respond accordingly. The responses consequently induce SPR signal changes as interactions between cells and molecules occurs [81,82,83]. In a study in 2007 by Yanase *et al.* [82], a large angle of resonance (AR) change in an SPR sensor was observed when RBL-2H3 rat mast cells and PAM212 cells (mouse keratinocyte cell lines) were cultured and activated on a sensor chip by an epidermal growth factor (EGF) or an antigen. In addition, the application of Mycalolide B and Toxin B (cell motility inhibitors) partially inhibited AR changes upon antigen stimulation; no cell movement or morphology changes were observed. These results suggest that AR changes reflect intracellular events other than cell-adhesion area size changes. SPR signal has been used to measure cell growth and size changes as well [84,85]. Exposure to non-isotonic stimulation can cause the cell volume to change. In particular, hypotonic stimulation results in SPR signal decrease, and the signal reverses to an equilibrium value after additional introduction of isotonic solutions.

The SPR technique plays a major role in cancer cell detection. The first approach is based on cancer biomarker production employing monoclonal antibodies immobilized on the sensor surface. The second approach directly measures cellular response for quantitative analysis. Cancer cells are seeded directly onto the sensing surface and cultured. Next, solutions containing stimuli are flowed over the cells, and their response is measured in real time by SPR. This strategy has been successfully applied to detect and diagnose malignant tumors cells, such as Chinese hamster ovary cells without the use of immunological labels [86]. Liu *et al.* designed an SPR biosensing system with anti-VEGF antibodies immobilized on the surface and living SKOV-3 ovarian cancer cells cultured on the flow cell chamber ceiling [87]. The cancer cells, in the activity promoting blood vessels growth, started to produce VEGF. Subsequently, these induced VEGF formed specific bindings with the antibody downward on the chip surface, and the SPR signal was recorded. The designed system allows the real-time detection of VEGF with sensitivity in a linear dynamic range of 0.1–2.5 μg∙mL^−1^. In 2006, our group described an integrative system using surface plasmon resonance imaging (SPRI) and a microwell gold protein chip [8]. With this system, we were able to carry out recombinant *E. coli* culture and the detection and purification of the expressed proteins on the chip [88]. The whole procedure included protein induction on the microwell chip, *E. coli* cell lysation (by adding lysozymes), purification of expressed glutathione S-transferase-fused green fluorescent protein (GST-GFP) by affinity interaction with gold surface chip, and finally the detection of expressed proteins using the SPRI system. The microwell chip was constructed using PDMS elastomer affixed on a gold surface forming a 36-well chip with a 5 uL sample volume for each well. This all-in-one system shows benefits in terms of time and labor saving, as it allows researchers to carry out the entire tedious protein process on a single chip. Thus, high-throughput expression and purification of recombinant proteins in drug development could benefit from the use of our system.

## 4. Immobilization of Biomolecules and Signal Amplification in SPR

### 4.1. Immobilization of Biomolecules

#### 4.1.1. Covalent Coupling

Covalent coupling is a commonly used immobilization method in the design of biosensor. To create covalent bonds, the reagents must activate the sensor chip surface to produce reactive species. Covalent coupling is then formed by the irreversible binding of reactive functional groups on the outside in a protein onto the activated surface. The chemical cross-linkers with spacer arms possess two functional groups; one is for covalent linkage to a surface, and the other functional group is for attachment to a biological molecule. Investigators have developed numerous covalent conjugation chemistries to produce protein-linker conjugates [89,90,91]. Several functional groups such as amine (-NH_2_), thiol (-SH), and aldehyde (-COOH) are most commonly used to covalently couple biomolecules to the solid support. Exposed amine (-NH2) groups on proteins can be coupled with various reactive intermediates such as *N*-Hydroxysuccinimide (NHS), aldehyde, and epoxide on the surface. In addition, thiol (-SH) groups present in cysteine residues can react and couple with a heterobifunctional crossliker such as a maleimide [92]. This thiol-maleimide reaction is a process that has been widely employed for the labeling of biomolecules. Another example of cysteine-mediated covalent conjugation is thiol-disulfide exchange coupling, which is usually derived by the coupling of two thiol groups [93]. Such covalently coupled-disulfide bonds are reversibly converted to thiols in the presence of reducing agents including dithiothreitol (DTT). Covalent coupling offers a stable, easy and ligand modification-free method for SPR surface functionalization. Nevertheless, in some cases, there are some drawbacks of the use of covalent coupling of biomolecules on a chemically-modified surface. First, covalent immobilization may involve chemical modification on the active sites of proteins which can potentially affect the analyte-binding activity. Second, the reactive functional groups can be blocked by nonspecific binding of proteins, and finally, an inappropriate blocking agents can inactivate the biomolecules.

#### 4.1.2. Sequence-Specific DNA-Directed Immobilization of Proteins

**Figure 5:**
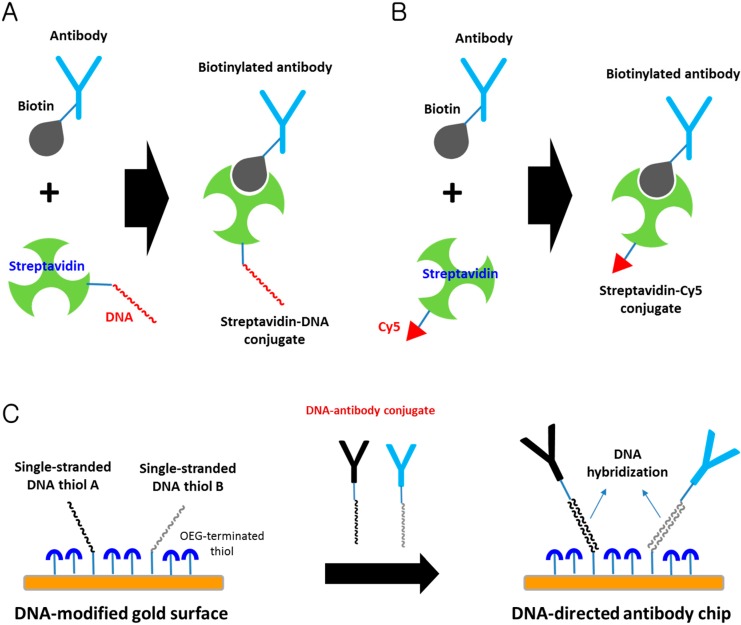
Schematic representation of DNA-directed immobilization. (**A**) Conjugation of four covalent conjugates (HA-HD) with the biotinylated antibodies anti-carcinoembryonic antigen (RAC), anti-ceruloplasmin (SAC), anti-complement-1-inactivator (SCI), and anti-lectin (GAL) to generate capture reagents A-RAC, B-SAC, C-SCI, and D-GAL. Adapted from [97]; (**B**) Conjugation of Streptavidin-Cy5 with biotinylated RAC, SAC, SCI and GAL for the use of a microscaled fluorescence immunoassay. Adapted from [97]; (**C**) DNA-directed immobilization of DNA-linked antibodies. c-A (Target sequence conjugated to anti-hCG): 5'-AGC GGA TAA CAA TTT CAC ACA GGA-3'; c-B (Target sequence conjugated to anti-hLH): 5'-AAC AGC TAT GAC CAT GAT TAC-3'; OEG, oligo (ethylene glycol). Adapted from [98].

Strategies for the sequence-specific DNA-directed immobilization of proteins (*i.e.*, antibodies, enzymes, peptides, *etc*.) include two main approaches, the immobilization of protein-DNA conjugates on a complementary DNA-modified surface and the immobilization of proteins harboring a DNA-binding domain on the cognate DNA-coated sensing layer. These DNA-directed immobilization techniques can orient proteins on a DNA chip surface in a manner that would allow the high binding affinity and specificity of proteins to their targets. Addressable biosensor chips using the site-direct immobilization of protein-DNA conjugates on a DNA-functionalized sensing layer have been developed [94,95,96]. Streptavidin-DNA conjugates have been used as molecular linkers in the DNA-directed immobilization of biotinylated antibodies on the chip surface (Figure 5A,B) [97]. In addition, DNA-linked antibodies have been applied onto a gold surface modified with complementary DNA (Figure 5C) [98]. The drawback of using antibody-DNA conjugates for DNA-directed immobilization is that labeling of antibodies with DNA molecules by covalent modification may have significant effects on their functional properties, that is, their abilities to bind antigens.

Studies on the DNA-binding domain-mediated immobilization of proteins on a consensus DNA-modified surface have been reported [99]. Likewise, Jeong *et al.* adopted the site-specific immobilization of proteins using sequence-specific DNA-binding domains for developing a protein microarray, which is a high-throughput tool for proteomics [100]. One representative tool for the characterization of protein-DNA interactions is SPR sensing. Sequence-specific interactions of the c-Myb protein with its specific sequences of DNA [101], and the p53 transcription factor with its consensus DNA binding sequences using SPR techniques have been reported [65]. DNA-binding domain-mediated protein immobilization on a cognate DNA-coated thin layer has applications in the on-chip profiling of transcription factor binding sites as well as the on-chip screening of protein-protein interactions between DNA-binding proteins and their interactants.

#### 4.1.3. Affinity Interactions

The affinity capturing strategy is based on the modification of the sensor chip to capture special tag-conjugated proteins. Ligands are linked to the surface and function as bait, and they are used to attract targets, such as affinity tag-fused protein. In general, affinity interaction utilizes the specific interactions between ligands and affinity tags, providing the uniform and oriented immobilization of target proteins. One example is the interaction between biotin (vitamin B7) and streptavidin (tetrameric glycoprotein, 4 × 13 kDa), which has been widely used as a non-covalent affinity immobilization technique to bind biomolecules to the sensor chip surface. Streptavidin-tagged proteins can strongly and rapidly interact with a biotin-functionalized surface [102]. They are also used to bridge the biotin-coated layer and the biotinylated biomolecules [103]. Affinity fusion tags, such as glutathione S-transferase (GST) specific for glutathione, maltose binding protein (MBP) for amylose, hexahistidine (6ХHis) for Ni-NTA, and so on have been used in a wide range of biological applications including the detection, purification, and immobilization of recombinant proteins. DNA encoding an affinity tag can be genetically combined with the protein gene of interest. With affinity fusion strategies, the simple and fast analysis of recombinant proteins (*i.e*., GST-, MBP-, and 6ХHis-tagged proteins) expressed in *E. coli* has been carried out using an SPR imaging device [104]. To control the orientation of the immobilized antibody, genetically engineered protein G with various affinity tags such as 6ХHis [105], GST [106], elastin [107], and DNA molecules [108] has been developed. Even if the Ni-NTA-6ХHis linkage exhibits a weaker binding affinity than the biotin-streptavidin complex, hexahistidine-mediated affinity interaction based on the concept of immobilized-metal ion affinity chromatography (IMAC) is one of the most promising methods for the orientation-controlled immobilization of proteins, since the functional properties of the tagged proteins are only slightly hindered by the small size of 6ХHis tag. Instead, some tagged proteins with large size affinity tags, such as GST (~25.6 kDa) or MBP (~40.6 kDa) can cause considerable steric hindrance, which may lead to the loss of structural integrity and biological activity of the affinity fusion partners.

#### 4.1.4. S-Au Bond in Cysteine-Gold Clusters

**Figure 6:**
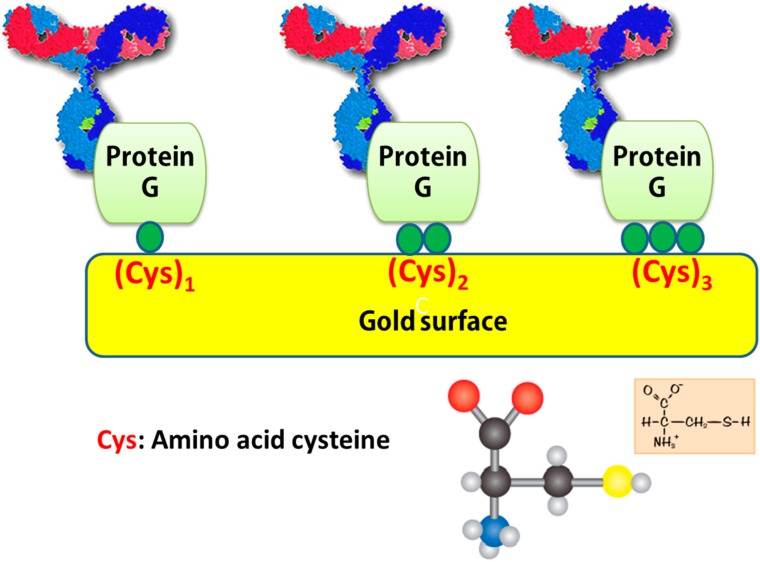
Schematic representation of direct immobilization of antibodies genetically modified with cysteines on a gold surface. A series of protein Gs containing two repeated IgG binding domains (B1 and B2) and a various numbers of cysteine amino acids, (Cys)_1_, (Cys)_2_, and (Cys)_3_, were genetically constructed, expressed/purified from *E. coli*, and subsequently analyzed using SPR biosensor. Adapted from [110].

Immobilization with unidirectional orientation is used to eliminate unevenly distributed complexes induced by random covalent coupling chemistries, thus developing a potent biosensor with high sensitivity and specificity. The high affinity between gold and sulfur-containing biomolecules is a well-known interaction concept for unidirectional immobilization. The orderliness in the SAM and molecular orientation can be achieved through S-Au binding, also referred to as the thiolated gold bond, involving the thiol (-SH) functional group. Cysteine residues have been widely used to immobilize proteins or peptides on an SPR gold surface for the purpose of controlled immobilization. With the advent of recombinant protein techniques, it is possible to express and purify a fusion protein containing cysteine amino acids with the ease of preparation of the cysteine-modified protein. [109]. The direct immobilization method has some important benefits such as speed, simplicity, favorable orientation, and increased functionality. Recently, the antibody-binding protein (ABP)-mediated immobilization of antibodies on a SPR surface has been carried out [110]. As shown in Figure 6, protein G, which specifically binds to the antibody Fc region, has been genetically modified with cysteine residues to immobilize antibodies on gold surfaces in an orientation-controlled manner, as antigen binding sites remain upright for undisturbed access of antigen to the antibodies, resulting in enhanced sensing performance. Well-oriented antibody immobilization based on cysteine-modified protein G does not hinder the active sites of the antibodies in response to an antigen; thus, the antibody activity on the biosensor surface is unaffected. This type of well-oriented, rather than randomly-oriented, immobilization demonstrates the enhanced antigen binding capacity of antibodies, allowing high sensitivity of antigen detection. Nevertheless, the use of protein G has a minor disadvantage that antibodies can be dissociated from the Fc region under certain conditions, such as low pH (2.0~3.0), leading to weaker signals.

### 4.2. SPR Signal Amplification

#### 4.2.1. Metal Nanoparticles

**Figure 7:**
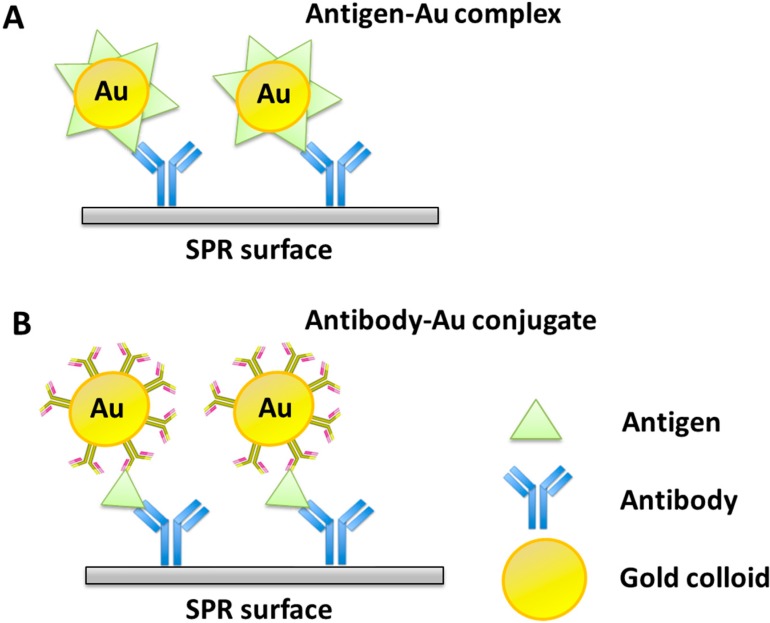
Schematic diagram of an immunochemical molecular recognition to illustrate the utility of Au colloid-enhanced biosensing. Two strategies for particle-enhanced SPR immunosensing are described: (**A**) Direct binding of the antigen-Au complex to an antibody-modified surface; (**B**) Antibody-modified surface followed by binding of a free antigen and then a secondary antibody-Au conjugate. Adapted from [112].

Metallic nanoparticles, especially gold nanoparticles (Au NPs), have been widely used to improve SPR-based sensing performance over the last decade. They are employed both in sensing surface enhancement and as amplification tags. Nanoparticle amplification tags are based on the fact that, upon addition of linked Au NPs, a pseudo mass increase of analyte is induced. This mass change finally produces higher refractive index changes on the SPR surface. In the first such work by Lyon and colleagues [111], colloidal gold particles of various sizes (30–59 nm in diameter) were used to coat a 47 nm-thick Au film. The results demonstrated that with increasing particle size, the limit of detection (LOD) and the maximum level of quantitation both decreased. In another work [112], Lyon *et al.* conjugated anti-human immunoglobulin (h-IgG) to Au NPs for a sandwich immunoassay to detect h-IgG. It was observed that as antibody-Au colloid conjugate was added, the signal was significantly amplified by as much as 25 times. A 6.7 pM detection limit of h-IgG was achieved using this method (Figure 7). In addition to Au NPs, other metal nanoparticles including Ag NPs, SiO2 NPs [113], Pd NPs [114], and Pt NPs [17] have also been applied to increase SPR sensitivity in various biomolecular detection applications.

#### 4.2.2. Magnetic Nanoparticles

**Figure 8:**
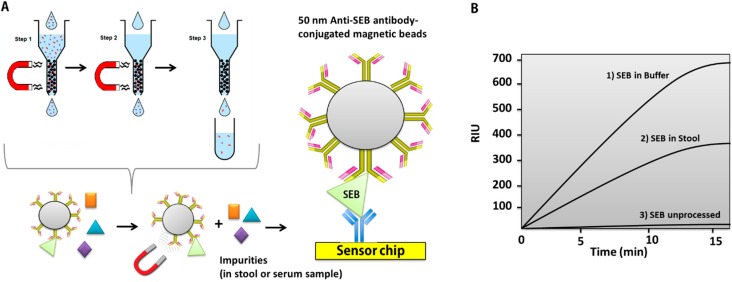
Strategy for detecting SEB in buffer and stool using antibody-coated magnetic nanobeads. (**A**) Steps for processing samples with colloidal immunomagnetic beads (antibody-coated superparamagnetic nanobeads). Anti-SEB antibody-modified nanobeads were mixed with solutions containing the target antigen (SEB) prior to the SPR measurement; (**B**) SPR detection of staphylococcal enterotoxin B (SEB) in buffer (663 RIU), in stool (365 RIU), and unprocessed (7 RIU). Adapted from [115].

The use of magnetic nanoparticles (MNPs) has a history of a few decades in bioseparation but not long in bioassays. These materials have attracted a great deal of attention due to their advantages, such as higher surface-to-volume ratio, minimum disturbance to attached biomolecules, faster binding rates, higher miscibility, and higher specificity. Compared to other plasmonic nanoparticles, MNPs are more cost effective. Thus, MNPs could facilitate SPR surface immobilization and sample precipitation by the use of a magnetic field [115]. In 2009, Soelbert *et al.* developed a method for the rapid and sensitive detection of Staphylococcal enterotoxin B (SEB) using antibody-coated magnetic nanobeads. The magnetic characteristics of the beads were utilized for SEB purification and concentration from real complex matrices before the samples were allowed to flow into their miniature SPR system (Figure 8). An LOD of 100 picograms/mL for SEB was achieved in both buffer and stool samples.

#### 4.2.3. Carbon-Based Nanomaterials

**Figure 9:**
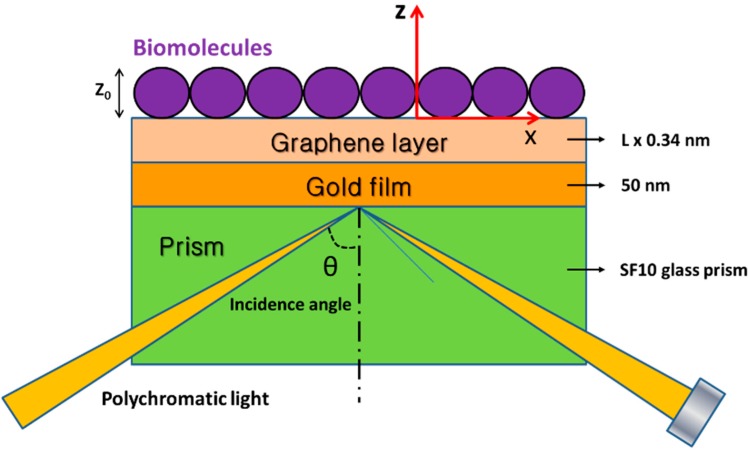
The configuration of the proposed graphene-on-gold surface plasmon resonance biosensor based on generalized N-Layer model, where the gold film is deposited on top of a SF10 glass prism. A polychromatic light wave passes through the prism and is internally reflected on the prism-gold interface, creating an evanescent wave which penetrate the metal film (50 nm) and propagate along the x direction with propagation constant. The light propagation constant matches the surface plasmon polariton (SPP) propagation constant across the interface by controlling the incident angle θ. Plots of totally reflected intensity *versus* incident angle yield a peak, which is known as SPR angle. The graphene-on-gold surface plasmon resonance: prism | Au (50 nm) | graphene (L × 0.34 nm) | sensing medium, where L is the number of graphene layers, and Z_0_ = 100 nm is the thickness of the biomolecule layer. Adapted from [116].

Nanomaterials in this group, such as grapheme carbon nanotubes have also been employed to assist SPR biosensor signal enhancement. Since the discovery of graphene in 2004, researchers have developed an enormous number of graphene applications in biomolecular detection and analysis. In 2010, study on SPR signal amplification using grapheme-coated gold sensing film was introduced [116]. Wu *et al.* modified the sensing substrate (conventionally gold film) by coating multiple graphene sheets over the gold thin film (Figure 9). Graphene is a known two-dimensional material with novel properties. One of the properties that make it useful in SPR signal enhancement is its super-speed electron transport at room temperature. The charge transfer from the surface of graphene to the surface of the Au thin film makes the sensing surface more sensitive to changes in the sensing medium. This is due to the strong excited electric field that exists on the surface of the Au thin film due to the graphene. The SPR signal was enhanced by as much as 25% when there were 10 graphene layers.

For optimal amplification, many authors have coupled multiple types of nanomaterials in their design so as to benefit from both sensing substrate enhancement and amplification tags. Examples include graphene oxide (GO) and gold nanorod (AuNR)–antibody conjugates [117], graphene oxide coupled with gold nanoparticles [118], multiple layers of graphene, and silicon on gold thin film [119]. In a publication by Lee [120], carbon nanotubes (CNTs) conjugated with a poly-clonal antibody were employed for the detection of human erythropoietin (EPO) and human granulocyte macrophage colony-stimulating factor (GM–CSF) in a sandwich assay format. The results showed that the signal obtained by this CNTs amplification tags were 30 times higher than those of a direct antibody-antigen scheme.

#### 4.2.4. Other Approaches

**Figure 10:**
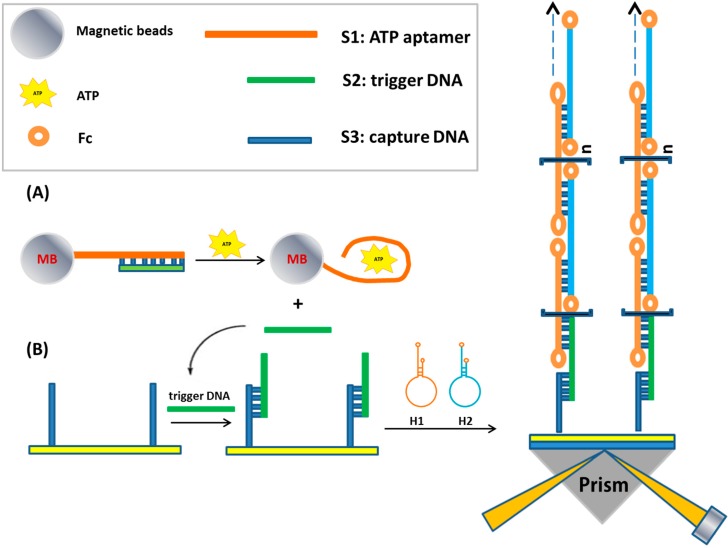
SPR assay for ATP detection amplified by DNA-based hybridization chain reaction (HCR). (**A**) The S1-magnetic bead conjugates were prepared by direct immobilization of an amine-modified ATP aptamer linked to an activated carboxylate group on magnetic beads through the EDC/NHS chemistry, and then hybridized with its complementary oligonucleotide S2; (**B**) The strategy of continuous SPR monitoring of trigger DNA on a DNA chip array. Following a magnetic separation from the solution, the gold surface, on which the thiolated capture DNA was immobilized through S-Au bond, was treated with the sample containing S2. The released S2 functioned as trigger DNA to bind to the cohesive end of H1 in the presence of ATP. Adapted from [123].

Despite the benefits of nanoparticles, there are also drawbacks of using them; for example, the synthesis and tuning steps are time and reagent-consuming. As SPR-based detection is heavily dependent on the mass change of the analytes in the sensing medium, researchers have tried to achieve signal amplification using biochemical approaches in combination with nanoparticles. Examples include rolling circle amplification (RCA) [121,122], hybridization chain reaction (HCR) [123,124,125], DNA manipulation driven [126,127,128], strand displacement amplification (SDA) [129,130], bio barcode techniques [131]. As an example, a multi-step amplification scheme using an integrative approach from hybridization chain reaction (HCR), magnetic beads, and strand displacement for the detection of DNA and ATP was developed recently by Li *et al.* (Figure 10) [123]. The strategy allows a linear detection range of DNA from 0.5 to 500 fM with a detection limit of 0.3 fM, which is quite impressive in comparison to other techniques. Furthermore, the strategy allows surface regeneration without loss of specificity (94.2% after 5 regenerations), and it could also be used to detect ATP in the range of 1.0 to 5000 nM with an LOD of 0.48 nM.

## 5. Recent Advances of SPR Technology

Even though SPR imaging (SPRI) has been considered as a powerful tool for monitoring or screening of biomolecular binding events in a label-free manner, SPRI using the prism configuration remains inherent limitations, which come from the physical constraint of the prism as it limits the numerical aperture (NA) and magnification of an imaging system, thus providing poor spatial resolution. For example, a SPR system which uses a 0.1 NA prism substrate could only give ~3 µm imaging resolution of the lens at a wavelength of 633 nm. In addition, in prism-type SPR, due to the relative movement between sample and camera, acquired images is caused to move and suffered from a distortion by the prism when the incident angle scanning is performed. This restraint entangles the data analysis and lowers the spatial resolution of the obtained SPR curve.

**Figure 11:**
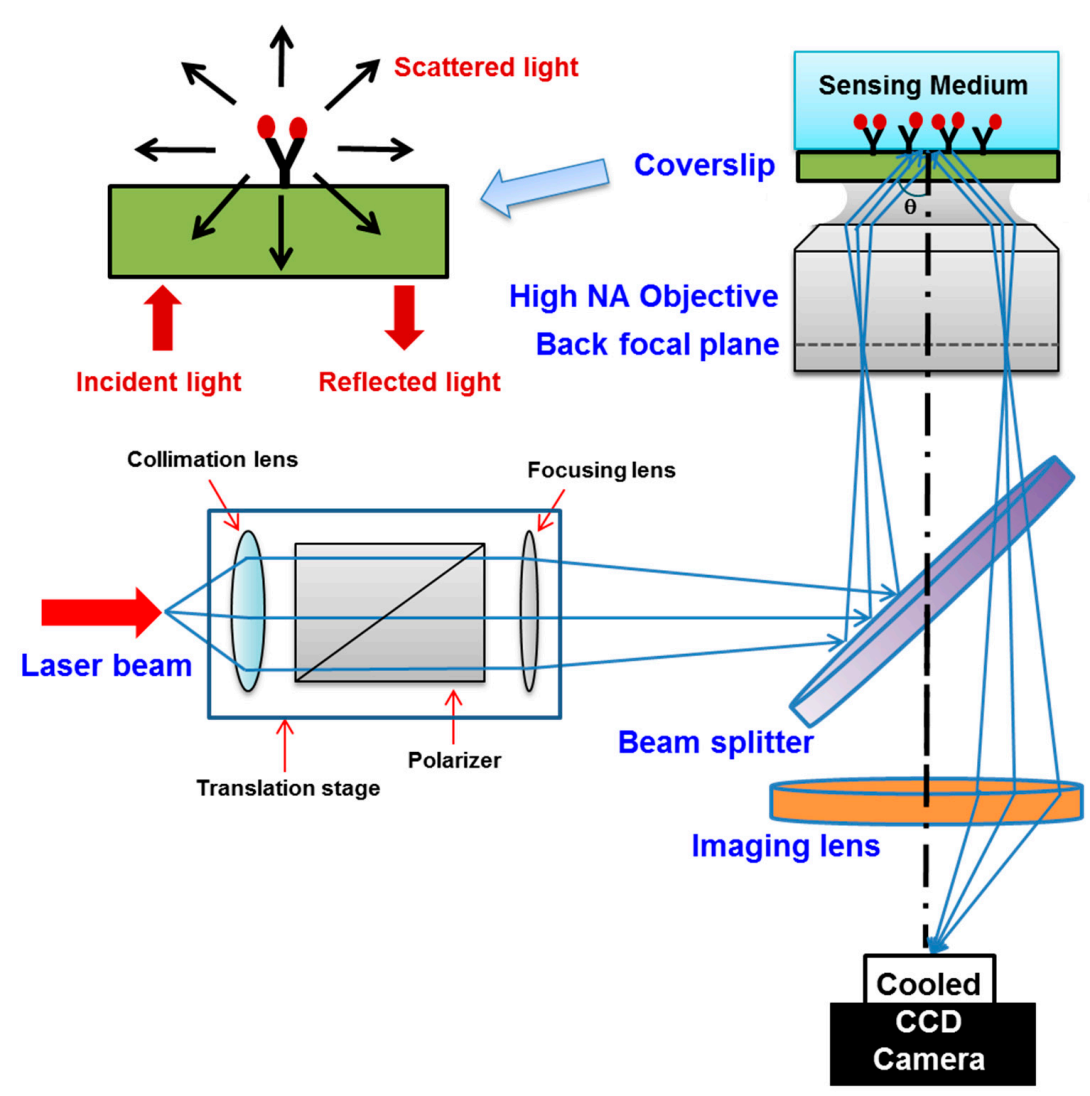
General principle of the objective-type SPRI setup. The optical configuration of the SPR microscope. Adapted from [133].

Recently, an alternative approach so-called high resolution SPR imaging that could correct all of the above addressed problems and offer a spatial resolution near the optical diffraction limit of the incident light has been developed [132,133,134]. Firstly proposed in 2007 by Huang *et al.* [133], an optical microscope objective-based SPR system built on the basis of the Kretschmann configuration using a high numerical aperture oil immersion objective and an inverted microscope has been successfully exploited in some critical applications such as single molecule analysis, or optical mapping (Figure 11).

A major superiority of objective-type SPRI is that the sample and imaging optical paths are fixed throughout incident angle scanning. Thus, it permits a pixel-by-pixel tracking of the reflectivity in the SPR images. Each of these pixels accordingly produces a SPR curve and the image is framed using the SPR minimum angle information. Following this approach, variations such as inhomogeneity and unwanted interferences from the laser intensity could be significantly removed by using a homogeneous referencing surface. More importantly, the divergence in incident angles at different spots of the image field caused by the abnormality of the objective lens could be corrected as well. Firstly, the use of a high NA and high magnification imaging system could provide a diffraction limit resolution (~300 nm) for the imaging optics [133]. Providing that SPR occurs at a larger angle than the critical angle, the immersion objective is chosen to have a NA (such as an Olympus 1.65 NA objective) which is larger than the refractive index of the medium. Consequently, when the incident light is transferred to the objective back aperture edge, it will arrive at the sample surface at an angle that is greater than the critical angle. Additionally, the angle coverage is also increased by this high NA objective. Secondly, compared to the angle scanning mode in a conventional SPR measurement, the use of an objective lens could simplify the system design by converting the rotational motion of the incident light at the sample into linear motion of the stage. Hence, it helps improve the overall mechanical performance of the system.

Taking advantage of the advanced SPRI new capabilities, researchers have successfully employed this high-spatial resolution SPR in imaging and detection of single DNA molecules [135], virus [136], cells [137]. Plus, single cell-substrate interactions mapping [137] and direct binding kinetics of proteins-cell membrane proteins mapping [138] were also made possible using this innovative system. The recent advances in plasmonic imaging technique using high-resolution surface plasmon resonance microscopy (SPRM) approach would undoubtedly have extensive impacts on the quantitative analysis of intracellular dynamics in live cells, single molecule analysis and the studies of the biological activities of membrane proteins as well as the discovery of new drugs that target membrane proteins.

## 6. Conclusions

SPR-based biosensor applications extend beyond the scope of this review paper. The SPR technique has been proven to be one of the most versatile frameworks for biosensors applications in the most concerning medical, biology, environment, and food safety areas. SPR biosensors provide excellent analytical performance in terms of high sensitivity, fast response, LOD and reproducibility with its label-free, real-time approach. However, one challenge for SPR technology, which was raised in 2008 by Schasfoort and Schuck about its suitability for point-of-care (POC) diagnostics [139], has not been completely solved. The inherent drawback of SPR lies in the interference of non-specific bindings to the outcome signals. Additionally, the kinetic rate and equilibrium constants data for biomolecular interactions obtained by SPR biosensors have low significance in POC analysis. Given the growing trend of applying biosensors in point-of-care testing (POCT), which is often performed by using portable and hand-held devices, SPR technology is one of the most promising tools because it is continuously progressing and evolving to be more suitable for user-friendly hand-held devices [69,140,141]. POCT is an important issue in settings where timing is critical (*i.e.*, emergency care or disease outbreaks). To meet this growing need for SPR devices that can be deployed in the field, every effort is being made to build more affordable, more accessible, and more applicable SPR sensor products. Clearly, a portable SPR system offers s strong potential for POCT applications related to security and defense against bioterrorism by detecting pathogenic species and identifying toxins in water in areas with no access to laboratory facilities. However, current SPR instruments are still bulky and costly, and this remains as an obstacle to the commercialization of SPR technology. In the coming years, more research on the development of innovative chip chemistry and antifouling strategies in combination with amplification schemes and miniaturization are needed to make SPR an irreplaceable tool for routine clinical analysis and POC diagnostics.
